# HCV cure: an appropriate moment to reduce cannabis use in people living with HIV? (ANRS CO13 HEPAVIH data)

**DOI:** 10.1186/s12981-022-00440-9

**Published:** 2022-03-15

**Authors:** Tangui Barré, Patrick Mercié, Caroline Lions, Patrick Miailhes, David Zucman, Hugues Aumaître, Laure Esterle, Philippe Sogni, Patrizia Carrieri, Dominique Salmon-Céron, Fabienne Marcellin, the ANRS CO13 HEPAVIH Study Group, D. Salmon, L. Wittkop, P. Sogni, L. Esterle, P. Trimoulet, J. Izopet, L. Serfaty, V. Paradis, B. Spire, P. Carrieri, M. A. Valantin, G. Pialoux, J. Chas, I. Poizot-Martin, K. Barange, A. Naqvi, E. Rosenthal, A. Bicart-See, O. Bouchaud, A. Gervais, C. Lascoux-Combe, C. Goujard, K. Lacombe, C. Duvivier, D. Neau, P. Morlat, F. Bani-Sadr, L. Meyer, F. Boufassa, B. Autran, A. M. Roque, C. Solas, H. Fontaine, D. Costagliola, L. Piroth, A. Simon, D. Zucman, F. Boué, P. Miailhes, E. Billaud, H. Aumaître, D. Rey, G. Peytavin, V. Petrov-Sanchez, A. Levier, R. Usubillaga, B. Terris, P. Tremeaux, C. Katlama, M. A. Valantin, H. Stitou, P. Cacoub, S. Nafissa, Y. Benhamou, F. Charlotte, S. Fourati, O. Zaegel, H. Laroche, C. Tamalet, P. Callard, F. Bendjaballah, C. Le Pendeven, B. Marchou, L. Alric, S. Metivier, J. Selves, F. Larroquette, V. Rio, J. Haudebourg, M. C. Saint-Paul, A. De Monte, V. Giordanengo, C. Partouche, A. Martin, M. Ziol, Y. Baazia, V. Iwaka-Bande, A. Gerber, M. Uzan, D. Garipuy, M. J. Ferro-Collados, F. Nicot, Y. Yazdanpanah, H. Adle-Biassette, G. Alexandre, J. M. Molina, P. Bertheau, M. L. Chaix, C. Delaugerre, S. Maylin, J. Bottero, J. Krause, P. M. Girard, D. Wendum, P. Cervera, J. Adam, C. Viala, D. Vittecocq, Y. Quertainmont, E. Teicher, C. Pallier, O. Lortholary, C. Rouzaud, J. Lourenco, F. Touam, C. Louisin, V. Avettand-Fenoel, E. Gardiennet, A. Mélard, A. Ochoa, E. Blanchard, S. Castet-Lafarie, C. Cazanave, D. Malvy, M. Dupon, H. Dutronc, F. Dauchy, L. Lacaze-Buzy, A. Desclaux, P. Bioulac-Sage, S. Reigadas, D. Lacoste, F. Bonnet, N. Bernard, M. Hessamfar, J, F. Paccalin, C. Martell, M. C. Pertusa, M. Vandenhende, P. Mercié, T. Pistone, M. C. Receveur, M. Méchain, P. Duffau, C. Rivoisy, I. Faure, S. Caldato, P. Bellecave, C. Tumiotto, J. L. Pellegrin, J. F. Viallard, E. Lazzaro, C. Greib, C. Majerholc, M. Brollo, E. Farfour, J. Polo Devoto, I. Kansau, V. Chambrin, C. Pignon, L. Berroukeche, R. Fior, V. Martinez, S. Abgrall, M. Favier, C. Deback, Y. Lévy, S. Dominguez, J. D. Lelièvre, A. S. Lascaux, G. Melica, F. Raffi, C. Allavena, V. Reliquet, D. Boutoille, C. Biron, M. Lefebvre, N. Hall, S. Bouchez, A. Rodallec, L. Le Guen, C. Hemon, D. Peyramond, C. Chidiac, F. Ader, F. Biron, A. Boibieux, L. Cotte, T. Ferry, T. Perpoint, J. Koffi, F. Zoulim, F. Bailly, P. Lack, M. Maynard, S. Radenne, M. Amiri, F. Valour, C. Augustin-Normand, C. Scholtes, T. T. Le-Thi, P. Chavanet M. Duong Van Huyen, M. Buisson, A. Waldner-Combernoux, S. Mahy, A. Salmon Rousseau, C. Martins, S. Galim, D. Lambert, Y. Nguyen, J. L. Berger, M. Hentzien, V. Brodard, M. Partisani, M. L. Batard, C. Cheneau, M. Priester, C. Bernard-Henry, E. de Mautort, P. Fischer, P. Gantner, S. Fafi-Kremer, F. Roustant, P. Platterier, I. Kmiec, L. Traore, S. Lepuil, S. Parlier, V. Sicart-Payssan, E. Bedel, S. Anriamiandrisoa, C. Pomes, M. Mole, C. Bolliot, P. Catalan, M. Mebarki, A. Adda-Lievin, P. Thilbaut, Y. Ousidhoum, F. Z. Makhoukhi, O. Braik, R. Bayoud, C. Gatey, M. P. Pietri, V. Le Baut, R. Ben Rayana, D. Bornarel, C. Chesnel, D. Beniken, M. Pauchard, S. Akel, C. Lions, A. Ivanova, A.-S. Ritleg, C. Debreux, L. Chalal, J. Zelie, H. Hue, A. Soria, M. Cavellec, S. Breau, A. Joulie, P. Fisher, S. Gohier, D. Croisier-Bertin, S. Ogoudjobi, C. Brochier, V. Thoirain-Galvan, M. Le Cam, M. Chalouni, V. Conte, L. Dequae-Merchadou, M. Desvallees, C. Gilbert, S. Gillet, R. Knight, T. Lemboub, F. Marcellin, L. Michel, M. Mora, C. Protopopescu, P. Roux, S. Tezkratt, T. Barré, T. Rojas Rojas, M. Baudoin, M. Santos V. Di Beo, M. Nishimwe

**Affiliations:** 1 Aix Marseille Univ, Inserm, IRD, SESSTIM, Sciences Economiques & Sociales de la Santé & Traitement de l’Information Médicale, ISSPAM, 35 boulevard Jean Moulin, 13005 Marseille, France; 2 Centre Hospitalier Universitaire (CHU) de Bordeaux, Pôle Médecine Interne, Service de Médecine Interne et Immunologie Clinique, Bordeaux Population Health Research Center UMR 1219, CIC-EC 1401, Université de Bordeaux, Pl. Amélie Raba Léon, 33000 Bordeaux, France; 3 Centre Hospitalier de Bourg-en-Bresse, Service d’Infectiologie, 900 Rte de Paris, 01012 Bourg-en-Bresse, France; 4 Réseau Ville-Hôpital, Service de Médecine Interne, Foch Hospital, 40 Rue Worth, 92150 Suresnes, France; 5 Centre Hospitalier de Perpignan, Service des Maladies Infectieuses et Tropicales, 20 Av. du Languedoc, 66000 Perpignan, France; 6 ISPED, Inserm, Bordeaux Population Health Research Center, Team MORPH3EUS, UMR 1219, CIC-EC 1401, Université de Bordeaux, 146 Rue Léo Saignat 11, Bordeaux, France; 7 Université Paris Descartes, 12 Rue de l’École de Médecine, 75006 Paris, France; 8 INSERM U1223, Institut Pasteur, 25 rue du Docteur Roux, 75015 Paris, France; 9 Hôpital Cochin, 27 Rue du Faubourg Saint-Jacques, 75014 Paris, France; 10 Service Maladies Infectieuses et Tropicales, AP-HP, Hôpital Cochin, 27 Rue du Faubourg Saint-Jacques, 75014 Paris, France

**Keywords:** Cannabis, Marijuana, HIV, Hepatitis C, Sustained virological response, HCV cure, Smoking, Behavioral changes

## Abstract

**Background:**

Thanks to direct-acting antivirals, hepatitis C virus (HCV) infection can be cured, with similar rates in HCV-infected and HIV–HCV co-infected patients. HCV cure is likely to foster behavioral changes in psychoactive substance use, which is highly prevalent in people living with HIV (PLWH). Cannabis is one substance that is very commonly used by PLWH, sometimes for therapeutic purposes. We aimed to identify correlates of cannabis use reduction following HCV cure in HIV–HCV co-infected cannabis users and to characterize persons who reduced their use.

**Methods:**

We used data collected on HCV-cured cannabis users in a cross-sectional survey nested in the ANRS CO13 HEPAVIH cohort of HIV–HCV co-infected patients, to perform logistic regression, with post-HCV cure cannabis reduction as the outcome, and socio-behavioral characteristics as potential correlates. We also characterized the study sample by comparing post-cure substance use behaviors between those who reduced their cannabis use and those who did not.

**Results:**

Among 140 HIV-infected cannabis users, 50 and 5 had reduced and increased their use, respectively, while 85 had not changed their use since HCV cure. Cannabis use reduction was significantly associated with tobacco use reduction, a decrease in fatigue level, paying more attention to one’s dietary habits since HCV cure, and pre-HCV cure alcohol abstinence (p = 0.063 for alcohol use reduction).

**Conclusions:**

Among PLWH using cannabis, post-HCV cure cannabis reduction was associated with tobacco use reduction, improved well-being, and adoption of healthy behaviors. The management of addictive behaviors should therefore be encouraged during HCV treatment.

## Introduction

In Western countries, AIDS is no longer the principal cause of death in people living with HIV (PLWH) [1–3]. Accordingly, HIV infection can be considered a chronic disease [4] associated with multiple comorbidities in aging people. In contrast, recent medical advances in hepatitis C virus (HCV) infection, specifically direct acting antivirals (DAA), provide a quick cure [5], and represent an important turning point in HIV–HCV co-infected people’s lives. This clinical change impacts quality of life [6–10] and foster behavioral changes [10, 11]. As psychoactive substance use is highly prevalent in HIV–HCV co-infected patients [12–15], we may expect HCV cure to impact substance use behavior. The benefits of cannabis and cannabinoid use for HIV infection management and less severe treatment side-effects, are widely recognized [16, 17], and PLWH frequently report their therapeutic use [18–22]. However, cannabis use may also promote pulmonary disease [23] and cognitive impairments in PLWH [24]. As cannabis use [14, 15, 21] and cannabis dependence [25] are frequent in HIV–HCV co-infected patients, it is important to explore changes in use after HCV cure. Using data from a cross-sectional survey embedded in the ANRS CO13 HEPAVIH cohort, we aimed to identify correlates of cannabis use reduction following HCV cure in HIV–HCV co-infected cannabis users, and to characterize persons who reduced their use.

## Material and methods

### Study design and data collection

ANRS CO13 HEPAVIH is an ongoing French national multicenter prospective cohort of HIV–HCV co-infected patients. Initiated in 2005, it investigates clinical and socio-behavioral issues surrounding HIV–HCV coinfection [26]. A total of 1859 patients followed in 29 hospital wards throughout metropolitan France were included in the cohort between October 2005 and March 2016, in three consecutive phases. Designed and implemented in accordance with the Declaration of Helsinki, the cohort and nested surveys were approved by the ethics committee of Cochin University Hospital in Paris. Patients provided written informed consent to participate.

A cross-sectional survey nested in the ANRS CO13 HEPAVIH cohort was conducted between February 2018 and May 2019 to document patient-reported outcomes, with a focus on perceived changes after HCV cure. All patients enrolled in the cohort and still followed-up in participating clinical centers at the time of the survey were offered to participate. A self-administered questionnaire (SAQ) collected data. The cohort is observational, therefore counselling and advice potentially received by participants depended solely on their physician.

The SAQ included questions related to sociodemographic characteristics, HCV transmission mode, recent substance use, cannabis dependence using the Cannabis Abuse Screening Test (CAST) [27], and reason for using cannabis (therapeutic motive or not). Other questions asked about changes since HCV cure in patients’ use of psychoactive substances (tobacco, cannabis, alcohol, other substances), physical activity, attention paid to dietary habits, and body weight gain. For questions documenting changes in substance use after HCV cure, respondents had to choose one answer among the following four: “No, nothing has changed”, “Yes, my use has decreased”, “Yes, my use has increased”, “Not concerned (no use)”.

### Study population

The population of the present study included HCV-cured cannabis users who participated in the cross-sectional survey and answered the SAQ item documenting perceived changes in cannabis use after cure. Patients who answered that they were not concerned by cannabis use were excluded from analyses.

### Statistical analysis

First, descriptive statistics were used to present the study population’s main characteristics. Comparisons were performed between patients who reported a reduction in cannabis use after HCV cure and those who did not (Chi-square test for categorical variables, Wilcoxon rank-sum test for continuous variables). Logistic regression models were then run to identify correlates of decreased cannabis use following HCV cure (study outcome). Socio-demographic variables and behavioral changes since HCV cure were tested as potential correlates. Only variables with a liberal p-value < 0.20 in the univariable analyses were considered eligible for the multivariable model. The final multivariable model was built using a backward stepwise procedure. The likelihood ratio test (p < 0.05) was used to define the variables to maintain in the final model.

Second, characteristics of substance use after HCV cure were compared between patients who reduced their cannabis use and those who did not using Chi-square tests. All statistical analyses were performed using SAS software version 9.4 for Windows (SAS, Cary, NC, USA).

## Results

Among the 448 survey participants, of the 421 HCV cured, two had no data on post-HCV cure changes in cannabis use. A total of 279 out of 419 patients reported no cannabis use (“Not concerned” answer) and were excluded from the analysis. The study population therefore comprised 140 individuals. Among them, 50 (35.7%) reported to have reduced their cannabis use after HCV cure, five had increased it, and 85 reported no change. The study sample mainly comprised men (74.3%), and median age was 55.7 years (Table 1). Six participants declared they quit cannabis.

**Table 1:** Study sample characteristics and factors associated with post-HCV cure cannabis use reduction (logistic regression model, ANRS HEPAVIH cohort, n = 140)

Variable	Total n (%)	Cannabis use reducers n (%)	Cannabis use non-reducers n (%)	p-value^1^	Univariable analyses			Multivariable analysis		
					OR	95% CI	p-value	aOR	95% CI	p-value
Gender				0.730						
Male	104 (74.3)	38 (76.0)	66 (73.3)		1.15	0.52–2.56	0.730			
Female	36 (25.7)	12 (24.0)	24 (26.7)		1					
Age (median, [IQR]) (years)	55.7 [53.0–58.5]	56.1 [53.2–58.6]	55.4 [52.9–58.5]	0.995	1.00	0.93–1.07	0.984			
HCV transmission mode				0.123			0.130			
Drug injection	91 (65.0)	38 (76.0)	53 (58.9)		1					
Sexual transmission	23 (16.4)	6 (12.0)	17 (18.9)		0.49	0.18–1.37	0.173			
Other	26 (18.6)	6 (12.0)	20 (22.2)		0.42	0.15–1.14	0.089			
Change in tobacco use^2^				< 0.001			< 0.001			0.014
No use	9 (6.4)	4 (8.0)	5 (5.6)		4.58	1.06–19.78	0.041	3.84	0.67–22.09	0.131
Reduction	57 (40.7)	35 (70.0)	22 (24.4)		9.11	3.96–20.96	< 0.001	4.32	1.58–11.78	0.004
No reduction	74 (52.9)	11 (22.0)	63 (70.0)		1			1		
Change in alcohol use^2^				< 0.001			< 0.001			0.003
No use	39 (27.9)	22 (44.0)	17 (18.9)		9.54	3.61–25.24	< 0.001	7.71	2.39–24.87	< 0.001
Reduction	34 (24.3)	20 (40.0)	14 (15.6)		10.54	3.85–28.81	< 0.001	3.32	0.94–11.72	0.063
No reduction	67 (47.9)	8 (16.0)	59 (65.6)		1			1		
Change in other substance use^2^				0.004			0.018			
No use	108 (77.1)	35 (70.0)	73 (81.1)		1.20	0.43–3.35	0.730			
Reduction	11 (7.9)	9 (18.0)	2 (2.2)		11.25	1.86–68.13	0.008			
No reduction	21 (15.0)	6 (12.0)	15 (16.7)		1					
Changes in physical activity^2^				0.238			0.244			
Stable	85 (60.7)	26 (52.0)	59 (65.6)		1					
Increase	39 (27.9)	16 (32.0)	23 (25.6)		1.58	0.72–3.47	0.256			
Reduction	16 (11.4)	8 (16.0)	8 (8.9)		2.27	0.77–6.70	0.138			
Changes in fatigue level^2^				0.021			0.023			0.073
Stable	63 (45.0)	15 (30.0)	48 (53.3)		1			1		
Reduction	63 (45.0)	30 (60.0)	33 (36.7)		2.91	1.36–6.23	0.006	3.12	1.15–8.46	0.025
Increase	14 (10.0)	5 (10.0)	9 (10.0)		1.78	0.52–6.13	0.362	2.95	0.61–14.15	0.177
Changes in dietary habits^2^				< 0.001			< 0.001			0.045
Stable	92 (65.7)	21 (42.0)	71 (78.9)		1			1		
Paying more attention	40 (28.6)	25 (50.0)	15 (16.7)		5.64	2.52–12.59	< 0.001	3.33	1.22–9.14	0.019
Paying less attention	8 (5.7)	4 (8.0)	4 (4.4)		3.38	0.78–14.69	0.104	3.01	0.48–18.79	0.237
Change in body weight ^2^				0.073			0.078			
No change or reduction	84 (60.0)	24 (48.0)	60 (66.7)		1					
Increase < 5 kg	33 (23.6)	14 (28.0)	19 (21.1)		1.84	0.80–4.25	0.153			
Increase ≥ 5 kg	23 (16.4)	12 (24.0)	11 (12.2)		2.73	1.06–7.02	0.038			

aOR: adjusted odds ratio; HCV: hepatitis C virus; IC: confidence interval; IQR: interquartile range

^1^Chi-square (categorical variables) or Wilcoxon rank-sum test (continuous variables)

^2^Self-reported post HCV-cure changes

Post-HCV cure decrease in cannabis use was associated with tobacco use reduction, pre-HCV cure alcohol use abstinence (p = 0.063 for alcohol use reduction), a decrease in fatigue level and paying more attention to one’s dietary habits (Table 1).

After HCV cure, regular or daily cannabis use was reported by most patients (54.8%), recreational use being predominant (59.5% of patients). No patient was at high risk of cannabis dependence (Table 2).

**Table 2:** Characteristics related to psychoactive substance use according to post-HCV cure reduction in cannabis use (cross-sectional survey nested in the ANRS CO13 HEPAVIH cohort, n = 140)

Variable	Total	Reduction in cannabis use after HCV cure		
		Yes	No	p-value^1^
Reason for using cannabis (n = 126)				0.324
Therapeutic	51 (40.5)	16 (34.8)	35 (43.8)	
Recreational only	75 (59.5)	30 (65.2)	45 (56.3)	
Recent substance injection^2^				0.454
No	139 (99.3)	50 (100.0)	89 (98.9)	
Yes	1 (0.7)	0 (0.0)	1 (1.1)	
Cannabis dependence (n = 129)^3^				0.904
No risk	64 (49.6)	22 (48.9)	42 (50.0)	
Low risk	65 (50.4)	23 (51.1)	42 (50.0)	
High risk	0 (0.0)	0 (0.0)	0 (0.0)	
Opioid substitution therapy (n = 133)				0.462
No	107 (80.5)	37 (77.1)	70 (82.4)	
Current therapy	26 (19.6)	11 (22.9)	15 (17.7)	
Cannabis use frequency (n = 126)				< 0.001
Never	6 (4.8)	6 (13.0)	0 (0.0)	
Sometimes	51 (40.5)	29 (63.0)	22 (27.5)	
Regularly or daily	69 (54.8)	11 (23.9)	58 (72.5)	
Other substance use^4^				0.041
No	127 (90.7)	42 (84.0)	85 (94.4)	
One or more	13 (9.3)	8 (16.0)	5 (5.6)	
AUDIT-C score	2.5 [0–5]	2 [0–4]	3 [0–5]	0.245
Alcohol use^5^				0.068
Not at risk	78 (55.7)	33 (66.0)	45 (50.0)	
At risk	62 (44.3)	17 (34.0)	45 (50.0)	
Tobacco use (n = 138)				0.001
No current use	19 (13.8)	11 (22.9)	8 (8.9)	
1 to 5 cig/d	32 (23.2)	17 (35.4)	15 (16.7)	
6 to 10 cig/d	41 (29.7)	12 (25.0)	29 (32.2)	
More than 10 cig/d	46 (33.3)	8 (16.7)	38 (42.2)	

AUDIT-C: Alcohol Use Disorders Identification Test Concise; cig/d: cigarette per day

^1^Chi-square (categorical variables) or Wilcoxon rank-sum test (continuous variables)

^2^In the previous 4 weeks

^3^Cannabis dependence assessed by Cannabis Abuse Screening Test [27]. A score < 3 defined ‘no risk’, a score ≥ 3 and < 7 defined ‘low risk’, and a score ≥ 7 defined ‘high risk’

^4^Any use of other substances (cocaine, heroin, crack, ecstasy, street Subutex, amphetamines, LSD, cathinone) in the previous 4 weeks

^5^At-risk use was defined as an AUDIT-C score ≥ 4 for men and ≥ 3 for women [42]

Those who reduced their cannabis consumption were more likely to use the drug less frequently, to have recently used other psychoactive substances (excluding alcohol and tobacco) and to smoke fewer tobacco cigarettes after HCV cure (p = 0.068 for alcohol use) (Table 2).

## Discussion

In this study, approximately one third of PLWH reduced their cannabis use after being cured of HCV. This reduction was associated with a reduction in tobacco use, pre-HCV cure alcohol abstinence, a decrease in fatigue level, and paying greater attention to one’s diet.

These results confirm previous findings that HCV cure is accompanied by behavioral changes, including changes in substance use [10, 11]. However, data on these changes are scarce [28], particularly in the HCV cure era, and especially for cannabis use, which is highly prevalent and partly motivated by therapeutic goals in PLWH.

Concomitant reduction in tobacco use is important for PLWH who reduce their cannabis use, as they are highly exposed to tobacco-related harms, a major morbidity and mortality risk factor in this population [29–31]. Cannabis and tobacco use frequently co-occur [32], especially in Europe [33]. Moreover, both drugs seem to reinforce each other [34, 35]. Accordingly, cannabis use impairs the chances of tobacco cessation [36], including in HIV–HCV co-infected people [15]. This phenomenon has also been documented for polysubstance use generally speaking [37, 38].

Our results suggest that HCV cure is an appropriate moment to engage in addictive behavior management, especially using a holistic approach for all substances. Our findings also suggest that lifestyle modifications post-HCV cure may include dietary changes. This is in line with studies showing that HCV cure is associated with increased self-care [39, 40], ability and motivation to plan for the future, self-confidence, and empowerment [40, 41]. However, our results also suggest that a reduction in cannabis (and tobacco) use in the PLWH population does not translate into abstinence.

We did not find any association between a therapeutic motive for cannabis use and post-cure reduction. However, participants who reduced their use were more likely to have experienced a decrease in their level of fatigue. This result suggests that the level of therapeutic benefit which HCV cure brings may only lead to a marginal reduction. Having said that, we cannot exclude reverse causality whereby the decrease in fatigue is the consequence of reduced cannabis use.

One of the study’s main limitations is that it is based on self-reports. We also had no data on physicians’ attitude and counselling regarding substance use after HCV cure. Moreover, we were not able to take into account the time since HCV cure in our models, and therefore the persistence of the observed reductions in use. However, our results still provide clues about the potential of using HCV treatment as a teachable moment for addiction treatment in PLWH.

## Conclusion

Among cannabis users living with HIV, post-HCV cure cannabis reduction was associated with tobacco use reduction, and approached significance for alcohol use reduction. The management of addictive behaviors should be emphasized during HCV treatment, and further research is needed to explore the psychosocial mechanisms at play in smoking behaviors among PLWH, especially regarding cannabis use.

## Data Availability

The datasets generated and analysed during the current study are not publicly available due to personal data but are available from the corresponding author on reasonable request.

## Abbreviations

CAST: Cannabis abuse screening test
DAA: Direct acting antivirals
HCV: Hepatitis C virus
PLWH: People living with HIV
SAQ: Self-administered questionnaire

## References

[1] Sellier P, Hamet G, Brun A, Ponscarme D, De Castro N, Alexandre G (2019). Mortality of people living with HIV in Paris Area from 2011 to 2015. AIDS Res Hum Retroviruses.

[2] Nishijima T, Inaba Y, Kawasaki Y, Tsukada K, Teruya K, Kikuchi Y (2020). Mortality and causes of death in people living with HIV in the era of combination antiretroviral therapy compared with the general population in Japan. AIDS.

[3] Fontela C, Aguinaga A, Moreno-Iribas C, Repáraz J, Rivero M, Gracia M (2020). Trends and causes of mortality in a population-based cohort of HIV-infected adults in Spain: comparison with the general population. Sci Rep.

[4] Deeks SG, Lewin SR, Havlir DV (2013). The end of AIDS: HIV infection as a chronic disease. Lancet.

[5] Das D, Pandya M (2018). Recent advancement of direct-acting antiviral agents (DAAs) in Hepatitis C therapy. Mini Rev Med Chem.

[6] Bertino G, Ragusa R, Corsaro LS, Frazzetto E, Messina V, Inguscio L (2020). Improvement of health-related quality of life and psychological well-being after HCV eradication with direct-acting antiviral agents. Real life setting data of an Italian cohort valued by Hepatitis Quality of Life Questionnaire (HQLQv2). Health Psychol Res.

[7] Fagundes RN, de Ferreira LEVVC, de Pace FL (2020). Health-related quality of life and fatigue in patients with chronic hepatitis C with therapy with direct-acting antivirals agents interferon-free. PLoS ONE.

[8] Ohlendorf V, Schäfer A, Christensen S, Heyne R, Naumann U, Link R (2021). Only partial improvement in health-related quality of life after treatment of chronic hepatitis C virus infection with direct acting antivirals in a real-world setting-results from the German Hepatitis C-Registry (DHC-R). J Viral Hepat.

[9] Scheiner B, Schwabl P, Steiner S, Bucsics T, Chromy D, Aichelburg MC (2016). Interferon-free regimens improve health-related quality of life and fatigue in HIV/HCV-coinfected patients with advanced liver disease: a retrospective study. Medicine.

[10] Lions C, Laroche H, Zaegel-Faucher O, Ressiot E, Bregigeon S, Geneau de Lamarliere P (2020). Hepatitis C virus-microelimination program and patient trajectories after hepatitis C virus cure in an outpatient HIV clinical unit. Eur J Gastroenterol Hepatol.

[11] Caven M, Malaguti A, Robinson E, Fletcher E, Dillon JF (2019). Impact of Hepatitis C treatment on behavioural change in relation to drug use in people who inject drugs: a systematic review. Int J Drug Policy.

[12] Cacoub P, Dabis F, Costagliola D, Almeida K, Lert F, Piroth L (2015). Burden of HIV and hepatitis C co-infection: the changing epidemiology of hepatitis C in HIV-infected patients in France. Liver Int.

[13] Hagan H, Thiede H, Des Jarlais DC (2005). HIV/hepatitis C virus co-infection in drug users: risk behavior and prevention. AIDS.

[14] Brunet L, Moodie EEM, Rollet K, Cooper C, Walmsley S, Potter M (2013). Marijuana smoking does not accelerate progression of liver disease in HIV-hepatitis C coinfection: a longitudinal cohort analysis. Clin Infect Dis.

[15] Barré T, Mercié P, Marcellin F, Esterle L, Duvivier C, Teicher E (2021). HCV cure and Cannabis abstinence facilitate tobacco smoking quit attempts in HIV-HCV co-infected patients (ANRS CO13 HEPAVIH Cohort Study). AIDS Behav.

[16] Mack A, Joy J. MARIJUANA AND AIDS [Internet]. Marijuana as medicine? The science beyond the controversy. National Academies Press (US); 2000. 25077214

[17] Haney M, Gunderson EW, Rabkin J, Hart CL, Vosburg SK, Comer SD (2007). Dronabinol and marijuana in HIV-positive marijuana smokers caloric intake, mood, and sleep. J Acquir Immune Defic Syndr.

[18] D’Souza G, Matson P, Grady CD, Nahvi S, Merenstein D, Weber K (2012). Medicinal and recreational marijuana use among HIV-infected women in the Women’s Interagency HIV Cohort (WIHS), 1994–2010. J Acquir Immune Defic Syndr.

[19] Towe SL, Horton OE, Martin B, Meade CS (2018). A comparison of motivations for marijuana use in HIV-positive and HIV-negative adults. AIDS Behav.

[20] Furler MD, Einarson TR, Millson M, Walmsley S, Bendayan R (2004). Medicinal and recreational marijuana use by patients infected with HIV. AIDS Patient Care STDS.

[21] Fogarty A, Rawstorne P, Prestage G, Crawford J, Grierson J, Kippax S (2007). Marijuana as therapy for people living with HIV/AIDS: social and health aspects. AIDS Care.

[22] Costiniuk CT, Saneei Z, Salahuddin S, Cox J, Routy J-P, Rueda S (2019). Cannabis consumption in people living with HIV: reasons for use, secondary effects, and opportunities for health education. Cannabis Cannabinoid Res.

[23] Lorenz DR, Uno H, Wolinsky SM, Gabuzda D (2019). Effect of marijuana smoking on pulmonary disease in HIV-infected and uninfected men: a longitudinal cohort study. EClinicalMedicine..

[24] Towe SL, Meade CS, Cloak CC, Bell RP, Baptiste J, Chang L (2020). Reciprocal influences of HIV and Cannabinoids on the brain and cognitive function. J Neuroimmune Pharmacol.

[25] Hartzler B, Carlini BH, Newville H, Crane HM, Eron JJ, Geng EH (2017). Identifying HIV care enrollees at-risk for cannabis use disorder. AIDS Care.

[26] Loko M-A, Salmon D, Carrieri P, Winnock M, Mora M, Merchadou L (2010). The French national prospective cohort of patients co-infected with HIV and HCV (ANRS CO13 HEPAVIH): early findings, 2006–2010. BMC Infect Dis.

[27] Legleye S, Piontek D, Kraus L, Morand E, Falissard B (2013). A validation of the Cannabis Abuse Screening Test (CAST) using a latent class analysis of the DSM-IV among adolescents. Int J Methods Psychiatr Res.

[28] Knight R, Roux P, Vilotitch A, Marcellin F, Rosenthal E, Esterle L (2017). Significant reductions in alcohol use after Hepatitis C treatment: results from the French ANRS CO13-HEPAVIH cohort: significant reductions in alcohol use. Addiction.

[29] Calvo M, Laguno M, Martínez M, Martínez E (2015). Effects of tobacco smoking on HIV-infected individuals. AIDS Rev.

[30] Helleberg M, May MT, Ingle SM, Dabis F, Reiss P, Fätkenheuer G (2015). Smoking and life expectancy among HIV-infected individuals on antiretroviral therapy in Europe and North America. AIDS.

[31] Reddy KP, Parker RA, Losina E, Baggett TP, Paltiel AD, Rigotti NA (2016). Impact of cigarette smoking and smoking cessation on life expectancy among people with HIV: a US-based modeling study. J Infect Dis.

[32] Agrawal A, Budney AJ, Lynskey MT (2012). The co-occurring use and misuse of cannabis and tobacco: a review. Addiction.

[33] Hindocha C, Freeman TP, Ferris JA, Lynskey MT, Winstock AR (2016). No smoke without tobacco: a global overview of cannabis and tobacco routes of administration and their association with intention to quit. Front Psychiatry.

[34] Panlilio LV, Zanettini C, Barnes C, Solinas M, Goldberg SR (2013). Prior exposure to THC increases the addictive effects of nicotine in rats. Neuropsychopharmacology.

[35] Ponzoni L, Moretti M, Braida D, Zoli M, Clementi F, Viani P (2019). Increased sensitivity to Δ9-THC-induced rewarding effects after seven-week exposure to electronic and tobacco cigarettes in mice. Eur Neuropsychopharmacol.

[36] Voci S, Zawertailo L, Baliunas D, Masood Z, Selby P (2020). Is cannabis use associated with tobacco cessation outcome? An observational cohort study in primary care. Drug Alcohol Depend.

[37] McCabe SE, West BT (2017). The three-year course of multiple substance use disorders in the United States: a national longitudinal study. J Clin Psychiatry.

[38] Crummy EA, O’Neal TJ, Baskin BM, Ferguson SM (2020). One is not enough: understanding and modeling polysubstance use. Front Neurosci.

[39] Batchelder A, Peyser D, Nahvi S, Arnsten J, Litwin A (2015). “Hepatitis C treatment turned me around:” psychological and behavioral transformation related to Hepatitis C treatment. Drug Alcohol Depend.

[40] Torrens M, Soyemi T, Bowman D, Schatz E (2020). Beyond clinical outcomes: the social and healthcare system implications of hepatitis C treatment. BMC Infect Dis.

[41] Williams BE, Nelons D, Seaman A, Witkowska M, Ronan W, Wheelock H (2019). Life projects: the transformative potential of direct-acting antiviral treatment for hepatitis C among people who inject drugs. Int J Drug Policy.

[42] Bradley KA, DeBenedetti AF, Volk RJ, Williams EC, Frank D, Kivlahan DR (2007). AUDIT-C as a brief screen for alcohol misuse in primary care. Alcohol Clin Exp Res.

